# Levels of DNA cytosine methylation in the *Drosophila* genome

**DOI:** 10.7717/peerj.5119

**Published:** 2018-07-02

**Authors:** Saniya Deshmukh, VK Chaithanya Ponnaluri, Nan Dai, Sriharsa Pradhan, Deepti Deobagkar

**Affiliations:** 1 Molecular Biology Research Laboratory; Department of Zoology (Centre for Advanced Studies), Savitribai Phule Pune University (formerly University of Pune), Pune, Maharashtra, India; 2 New England Biolabs, Ipswich, MA, United States of America

**Keywords:** DNA methylation, 5-methylcytosine, *Drosophila*, UHPLC-QQQ

## Abstract

Insects provide an accessible system to study the contribution of DNA methylation to complex epigenetic phenotypes created to regulate gene expression, chromatin states, imprinting and dosage compensation. The members of genus *Drosophila* have been used as a model system to study aspects of biology like development, behaviour and genetics. Despite the popularity of *Drosophila melanogaster* as a genetic and epigenetic model organism, DNA methylation studies are limited due to low levels of genomic 5-methylcytosine. Our study employs a sensitive liquid chromatography-mass spectrometry (LCMS) based method to quantify the levels of 5-methylcytosine from the genomic DNA in different members of the genus *Drosophila*. Our results reveal that, despite being phylogenetically related, there is a marked variation in the levels of 5-methylcytosine between the genomes of the members of genus *Drosophila*. Also, there is a change in the genomic levels of 5-methylcytosine through each life cycle stage of holometabolous development in *D. melanogaster*.

## Introduction

The epigenome of an organism constitutes histone modifications (Zentner & Henikoff, 2013), non-coding RNA molecules (Busto et al., 2015; Stuwe, Tóth & Aravin, 2014) and nucleotide modifications (Achwal, Ganguly & Chandra, 1984; Achwal, Iyer & Chandra, 1983; Gowher, Leismann & Jeltsch, 2000; Zhang et al., 2015). These modifications can together or independently influence the regulation of gene expression and conserve the energy resources by managing functional conformation of the genome (Zee et al., 2016). The epigenetic changes are important in the insect taxon which commonly exhibits polyphenisms (Simpson, Sword & Lo, 2011). Unlike mammals and plants, the sparse methylation of gene bodies and transposons is characteristic of insect DNA methylation (Li-Byarlay, 2016). In social insects with the canonical DNMT1 and DNMT3A/3B methyltransferases, DNA methylation can be attributed to differential splicing, regulation of expression and histone occupancy, whereas very little is known in solitary insects which mostly possess only DNMT2 methyltransferase (Glastad, Hunt & Goodisman, 2014).

The members of genus *Drosophila* undergo holometabolous development beginning with the embryonic stage which eventually develops into an adult fly after passing through larval and pupal stages (Hartenstein, 1993). There are various changes observed in the DNA methylation patterns of model systems like the house mouse during their development from embryo to adulthood (Smith et al., 2012; Smith & Meissner, 2013). Some previous studies report the presence of low levels of 5-methylcytosine (5mC) and an active DNA methyltransferase in *Drosophila melanogaster* (Achwal, Ganguly & Chandra, 1984; Achwal, Iyer & Chandra, 1983; Gowher, Leismann & Jeltsch, 2000; Panikar et al., 2015). Recently, the presence of 5mC (less than 1%) in *D. melanogaster* genome was confirmed by 5mC-specific immunoprecipitation followed by bisulfite sequencing in stage 5 embryos (Takayama et al., 2014) and liquid chromatography/tandem mass spectrometry in adult stage (Capuano et al., 2014; Rasmussen et al., 2016). Bisulphite sequencing is a commonly used method for detection of genome-wide DNA methylation as it provides sequence context information of methylated cytosine residues. However, this method has limitations due to the lower concentration range, incomplete bisulphite conversion of unmethylated cytosines and misalignment of sequenced reads due to genomic repeats, telomeres and GC-rich regions (Warnecke et al., 2002). Using an alternative LCMS-based protocol, Capuano et al., 2014 have reported DNA methylation over a large range from 0.034% of cytosines methylated in a sample with females and males in equal proportion of wt/w118 *D. melanogaster* adults to 7.6% in liver tissue from *Mus musculus* and 14% from leaf tissue of *Arabidopsis thaliana*. The level of DNA methylation in *Drosophila* is 10–100 folds below the detection limit of bisulphite sequencing. This study established the advantage of an LCMS-based method to assess the levels of DNA methylation in systems like *Drosophila* with low levels of 5mC.

It is known that DNA methylation patterns change due to the life cycle stage, the tissue or cell type and the age of the specimen under consideration (Lokk et al., 2014). Independent studies mentioned above have demonstrated the presence of 5mC by different techniques in the embryo and adult stages of *D. melanogaster*. We have estimated the amount of 5mC in the genome of *D. melanogaster* (across all the life cycle stages) and other 11 species from genus *Drosophila* using ultra-high performance liquid chromatography/triple quadrupole mass spectrometry. Our analysis determines the change in the levels of 5mC during holometabolous development within a species and also between member species of genus *Drosophila.*

## Materials & Methods

### Sample preparation and DNA isolation

The dechorionated 8–14 h old embryos from a synchronised batch of flies, larvae from third instar stage, male and female pupae, adult male and female flies (one day old) were collected from the laboratory culture of Oregon-R strain maintained on corn-meal medium under standard conditions. These samples were washed in 70% ethanol and 1X PBS (to surface sterilise the larva and adult), then processed for DNA isolation. Briefly, the samples were homogenised after snap-chilling in liquid nitrogen and incubated at 65 °C with RNase A (12091–021) for 4 h; followed by standard phenol-chloroform extraction. Treatment with 1:2.5 parts of 5M potassium acetate and 6M lithium chloride to remove any traces of RNA was given followed by precipitation by iso-propanol. The DNA samples were dissolved in TE buffer. The samples for all the 12 species of genus *Drosophila* analysed were also obtained in parallel from UCSD Drosophila Species Stock Center, USA. The DNA samples were checked for integrity using an agarose gel and NanoDrop full-spectrum, UV-Vis spectrophotometers which ensure quality output.

### Ultra-high performance liquid chromatography/triple quadrupole mass spectrometry analysis (UHPLC/QQQ)

DNA (500 ng) was digested in 20 µL total volume with a NEB nuclease enzymes mix for 2 hr to obtain individual nucleosides. dU was spiked in as an internal control for all samples. The percentage of 5mdC/dC present in the sample was obtained by determining d5mC/dU and dU/dC values. The samples were resolved on a waters column (XSelect HSS T3 XP column (2.1  × 100 mm, 2.5 µm)) using ammonium formate and methanol buffer system on an Agilent 1290 UHPLC with 6490 QQQ Mass detector in MRM mode which is an established method for nucleoside detection (Estève et al., 2014). Similarly, the adult samples of 12 species obtained from UCSD *Drosophila* Species Stock Center, USA were analysed.

The samples were run in minimum biological triplicates and technical duplicates to ensure reproducible values; the multiple and pair-wise sample comparisons were made by Kruskal-Wallis with post hoc tests (Mann–Whitney U and Dunn’s test) and Mann–Whitney U tests respectively using PAST (Hammer, Harper & Ryan, 2001) and GraphPad Prism version 5 for Windows (GraphPad Software, La Jolla, CA, USA; http://www.graphpad.com/). The statistical tests ensured within and between group variance and effect of outliers (if any) were accounted for in the comparisons.

### Nucleotide counting and estimation of GC content for twelve genus *Drosophila*

The FlyBase is an extensive online sequence resource; encompassing fully sequenced genomes for twelve species of genus *Drosophila* which can be used for comparative genomic analyses (Crosby et al., 2006; Gramates et al., 2017). These are the twelve most commonly used, annotated *Drosophila* genomes. The latest whole genome sequences releases were downloaded from the FlyBase database. A Perl code was constructed and used for counting the number of individual nucleotides from each sequence fasta file and the percent GC was calculated. The written code was cross-checked with resource material available on Biostars (https://www.biostars.org/) and the veracity of the output count for the code was performed by comparing the nucleotide counts and genome length with the current release details of *D. melanogaster* genome. The UHPLC output counts methylated cytosines per 100 cytosines counted; this data was used to extrapolate the number of 5mC per haplogenome.

### Phylogenetic analysis for the for twelve genus *Drosophila*

18S rRNA gene sequences for all the twelve species of genus *Drosophila* were extracted from the NCBI repository. The phylogenetic tree was constructed using MEGA version 6 software (Tamura et al., 2013). The sequences were aligned using the Muscle multiple alignment option using the default parameters. The phylogenetic tree was constructed using the neighbour-joining (NJ) method with 1,000 bootstrapping.

### Identification of methyltransferases homologs in genus *Drosophila*

In order to identify the presence of a common methyltransferases protein present in the twelve members of genus *Drosophila*. The protein sequence of the only identified DNA methyltransferase, DNMT2 from *D. melanogaster*, was used as a query for the BLAST algorithm. The proteins from the other eleven members with 70–100% identity were selected. The selected twelve sequences were uploaded to the PROMALS3D server to generate a multiple sequence alignment and identification of secondary protein structures (Pei, Kim & Grishin, 2008).

## Results

In order to analyse the presence of DNA methylation in the members of genus *Drosophila* we first standardised the protocol for detection of 5mC detection in *D. melanogaster* using the UHPLC/QQQ.

The comparison of samples from the life cycle stages of *D. melanogaster* by Kruskal-Wallis (KW) test shows a significant difference between the levels of 5mC (KW, *p* = 0.001). The late stages of the embryo in our study represent the stages of development after cellularization and the germ band elongation has been completed. The embryonic stages from 12 to 16 used for representing the late embryos maintain the same general body plan across the later stages in the life cycle. These embryonic stages exhibit significantly high levels of 5mC amongst all the life cycle stages of *D. melanogaster*.

**Figure 1:**
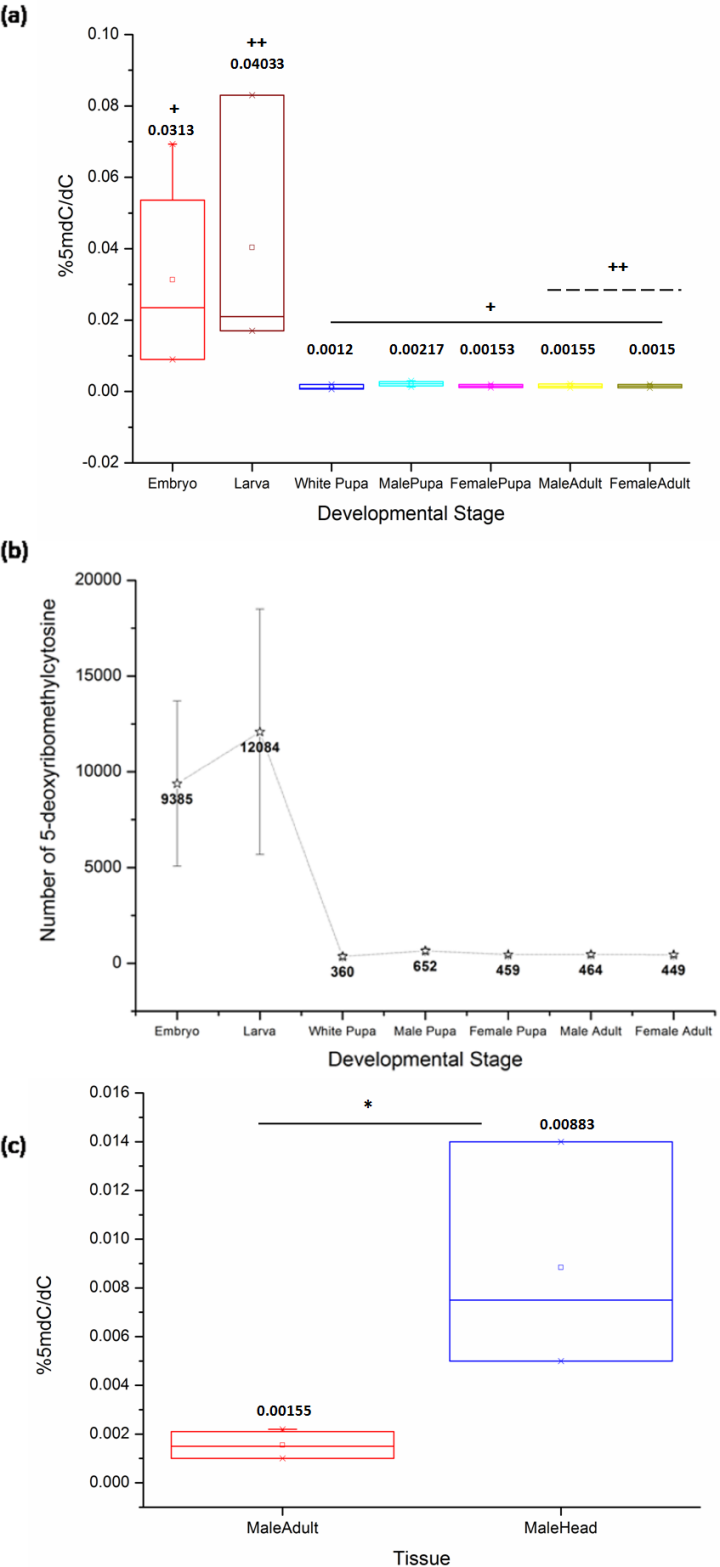
The percentage of 5mC per cytosine nucleotides across the developmental stages. (A) + indicates a significant difference between the embryo and other stages, whereas ++ indicates a significant difference between the larval and adult stages in both sexes (<0.05, *p* = 0.001). The comparison was significant between embryonic stages and white pupa (*p*-value = 0.04), male pupa (*p*-value = 0.02), female pupa (*p*-value = 0.04), adult male (*p*-value = 0.02) and female (*p*-value = 0.02). Additionally, there is a significant difference between the third instar larva and both sexes of the adult fly stage (*p*-value = 0.04). Kruskal-Wallis test with post hoc Mann-Whitney *U* test. (B) Representative change in the number of methylated cytosine in the *Drosophila* haplo-genome. (C) Difference in the levels of 5mC in the head tissue and the whole body of adult male *D. melanogaster*. * indicates significance of comparison (<0.05, *p* = 0.035, Mann–Whitney *U* test). All error bars represent SEM.

Also, there is a significant difference between DNA methylation of the third instar larva and both sexes of the adult stage (MW, *p* = 0.04). The ratio of %5mdC/dC does not show statistically significant difference amongst other stages in the life cycle (Fig. 1A) (Table 1, Supplementary File). There is a decrease in the levels of 5mC after metamorphic transition following the larval stage which is maintained throughout development. The early pupae or white pupae, the sexually distinct pupal and adult stages show similar levels of global DNA methylation. This indicates that there is no sex-specific difference in the levels of 5mC in age-matched samples of the same stage.

**Table 1:** The percentage of 5mC nucleotides per cytosine nucleotides counted in different developmental stages of *D. melanogaster*.

Developmental stage	% 5mdC/dC	Std. error
Late embryos	0.026	0.0137
3rd instar larva	0.025	0.0165
White pupa	0.001	0.0004
Male pupa	0.002	0.0003
Female pupa	0.001	0.0002
1d Male adult	0.001	0.0003
1d Female adult	0.001	0.0002

Using a combination of the current *D. melanogaster* genome release by FlyBase and our results with a Perl script to count the number of deoxyribonucleotides in the genome, we have extrapolated the number of modified cytosines in each developmental stage (details in Supplementary File, sheet 2). The number of methylated cytosines per copy of genome in each developmental stage and nucleotide composition of the fly genome are represented in (Fig. 1B).

Although the overall levels remain comparable, the changes in the local patterns of DNA cytosine methylation cannot be ruled out. The head tissue distinctly has a higher level of 5mC when compared to the whole body of the adult (MW, *p* = 0.035) (Fig. 1C). This indicates that in *Drosophila* methylation patterns can be rearranged and redistributed between different tissues. There is no significant difference between the heads of male and female adult *D. melanogaster* (Fig. S1). The presence of higher level of 5mC in the head also eliminates the possibility of methylation being detected due to the possible contamination from gut microflora or endosymbionts as a false positive in *Drosophila* genomic DNA.

**Figure 2:**
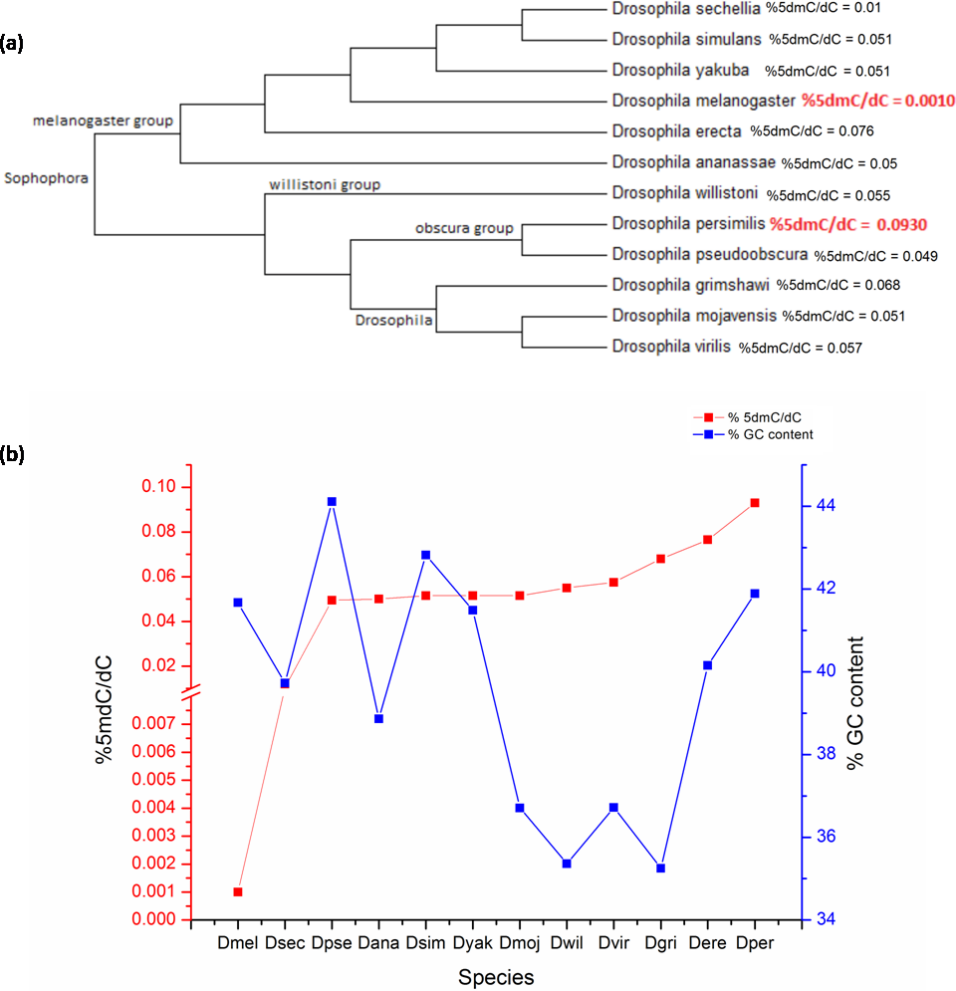
The percentage of 5mC nucleotides per cytosine nucleotides and the percentage of GC content in the genomes of members of *Drosophila* species. (A) Phyogenetic relationship between the members of genus *Drosophila*. The two species with the highest and the lowest levels of 5mC are indicated in red. (B) The left Y-axis represents percentage of 5mC nucleotides per cytosine nucleotides counted and the right *Y*-axis represents the percentage of GC content of the genome; the *X*-axis represents *D. melanogaster* (Dmel), *D. sechellia* (Dsec), *D. pseudoobscura* (Dpse), *D. ananassae* (Dana), *D. simulans (Dsim), *D. yakuba* (Dyak), *D. mojavensis* (Dmoj), *D. willistoni* (Dwil), *D. virilis* (Dvir), *D. grimshawi* (D.gri), *D. erecta* (Dere), *D. persimilis* (Dper)*. (< 0.05, *p* = 0.0217, KW test for comparison of means).

*D. melanogaster* is a member of the melanogaster group of the subgenus *Sophophora* and is one of 12 fruit fly species with completely sequenced genome for comparative genomics. There is no date available on the presence of DNA methylation in the other 11 species. In order to assess the distribution of 5mC across genus *Drosophila*; DNA from the adult stage of twelve prominent member species from subgenera *Drosophila* and *Sophophora* was analysed. The bootstrap consensus tree for all the species is shown to provide information on the most frequently appearing branch groupings using the 18S rRNA sequences (original NJ tree and divergence time in Figs. S2 and S3) (Fig. 2A). There is a significant difference between the values of the 5mC amongst the twelve-species analysed (*p* = 0.0217, KW). The members of subgenus *Drosophila* carry more 5mC in their genome when compared to the *Sophophora* (Stage & Eickbush, 2007). *Drosophila melanogaster* has the lowest while *Drosophila persimilis* has the highest levels of 5mC amongst the analysed genomes. Our results show that even closely related species show a difference in the levels of DNA methylation; *D. simulans* and *D. sechellia* are closely related species from the melanogaster group but have a substantial difference in levels of 5mC in their genome. The levels of 5mC observed across these species do not seem to show any correlation with their phylogenetic relatedness. The members of the melanogaster group posses a diverse profile of global DNA methylation. The lowest level DNA methylation value of 0.001% 5dmC/dC is observed in the genome of *D. melanogaster* and *Drosophila erecta* genome has the 0.0765% 5dmC/dC between the members of the melanogaster group. However, the subgenus *Drosophila* possesses a comparable distribution of 5mC among its members.

**Figure 3:**
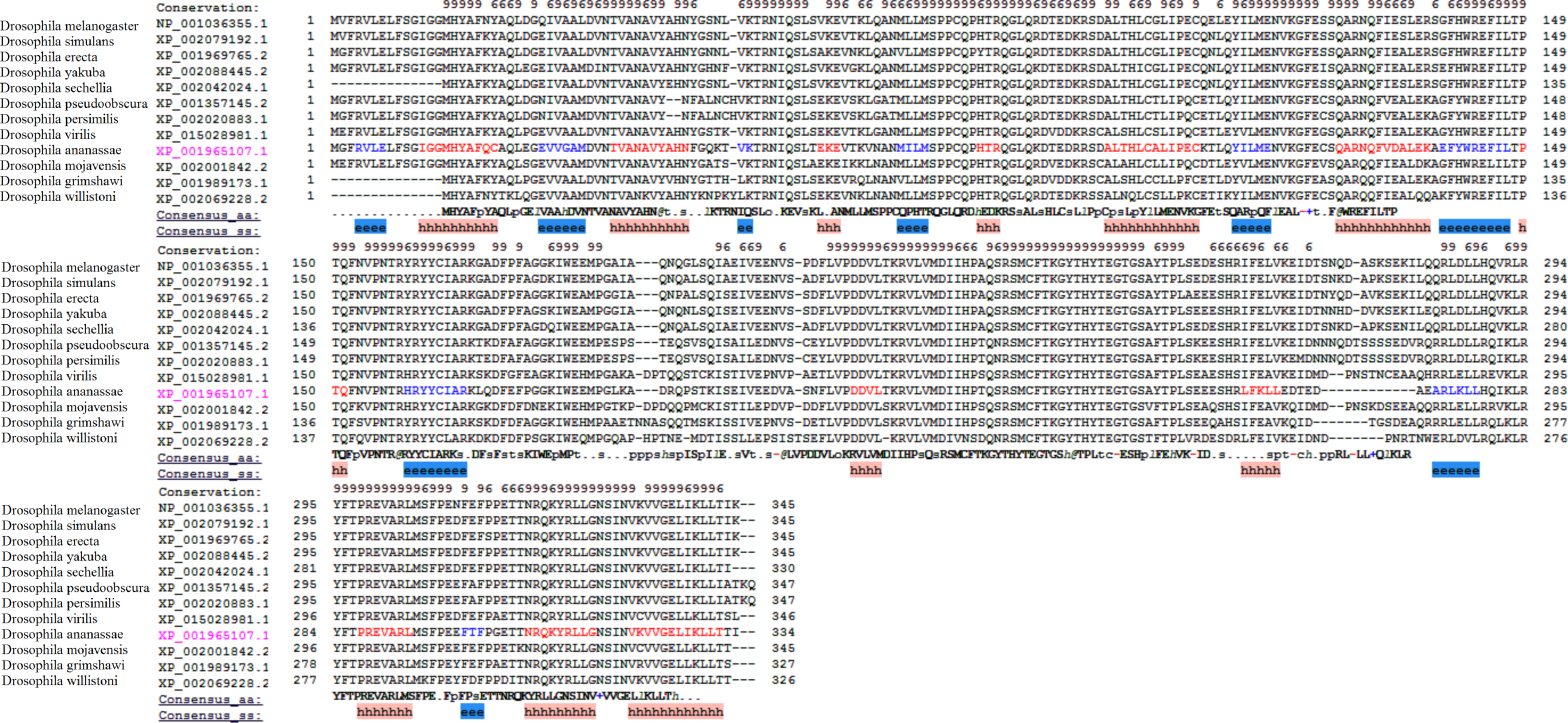
The multiple alignments of protein sequences retrieved from the NCBI repository with conserved domain architecture with DNMT2 methyltransferase in *D. melanogaster*. The ‘e’ and ‘h’ regions represent beta strand and alpha helix consensus secondary structures from the residues in the twelve proteins.

From the available genome releases and our data, we have compared the GC content, the values of 5mC and C present and %5mdC/dC for the twelve species (Fig. 2B, Table 2, Figs. S1 and S2). *Drosophila grimshawi* and *Drosophila willistoni* possess the lowest number of cytosines in their genomes while *Drosophila pseudoobscura* has the highest level of cytosines. Despite the lowest GC content, *D. grimshawi* and *D. willistoni* have 0.068 and 0.055% 5dmC/dC which is comparable to 0.0495% 5dmC/dC in *D. pseudoobscura*. Likewise, with a vast difference in the number of 5mC per genome copy, *D. melanogaster* and *D. persimilis* have similar genomic GC content (Supplementary File, sheet 3).

**Table 2:** The 5mC count per haplogenome, percentage of 5mC nucleotides per cytosine nucleotides counted in genome and percent GC content for member species of genus *Drosophila*.

Species	Total number of 5mC/haplogenome	%5dmC/dC	%GC content
*D. melanogaster*	300	0.001	42
*D. sechellia*	3,472	0.010	40
*D. pseudoobscura*	16,675	0.049	44
*D. ananassae*	22,468	0.050	39
*D. simulans*	13,774	0.051	43
*D. yakuba*	17,696	0.051	41
*D. mojavensis*	18,327	0.051	37
*D. willistoni*	22,910	0.055	35
*D. virilis*	21,769	0.057	37
*D. grimshawi*	24,046	0.068	35
*D. erecta*	23,451	0.076	40
*D. persimilis*	36,691	0.093	42

*D. melanogaster* has been identified as one of the DNMT2 only organism, as there is an absence of any other de novo or maintenance methyltransferases (Lyko & Maleszka, 2011). DNMT2 protein from *D. melanogaster* as the query protein for the BLAST algorithm, similar protein was identified from the other analysed *Drosophila* species. The sequences with a high identity score between 70–100% and strong *E* value and at least 95% query coverage were filtered from the obtained results. The multiple sequence alignment of the extracted sequences similar to the DNMT2 of *D. melanogaster* show strongly conserved regions; they also bear identical secondary structures from the consensus residues aligned using PROMALS3D server (Fig. 3) (Pei, Kim & Grishin, 2008). The possibility of the existence of an unknown methyltransferase or a protein complex can be considered as studies in *D. melanogaster* embryos with DNMT2 knockout has been shown to have persistence of DNA methylation (Takayama et al., 2014).

## Discussion

HPLC-based analysis has shown the presence of 0.034% methylation in a mixed population of w^1118^ adult flies and 0.002% 5-methylcytosine/cytosine in adult females of *ore-R* lab strain in the genome of *D. melanogaster*. Also, there is a gene-specific distribution of 5mC methylation during *D. melanogaster* development (Panikar et al., 2017). Our current analysis reports a change in the levels of global DNA methylation using UHPLC/QQQ which allows detection of 5mC with greater accuracy as compared to previously used antibody-based techniques. The method used overcomes limitations of bisulfite-sequencing due to the low concentration of 5mC in *Drosophila* but does not provide sequence context information on DNA methylation. Hence, we are unable to extrapolate the location of the methylation with respect to annotated genomic features like the transposable elements, promoters, repeats etc.

DNA methylation is present in many insects like hymenopterans, lepidopteran, etc., but is much lower than in mammals and plants (Glastad, Hunt & Goodisman, 2014; Simola et al., 2013; Xiang et al., 2010). The functional importance of DNA methylation in insects is better understood in the eusocial systems like honey bees and wasps (Hunt et al., 2013). The existing evidence suggests that DNA methylation in the eusocial systems delegates the social order by means of alternative splicing or ploidy variation (Glastad et al., 2014; Lyko et al., 2010).

Most Dipterans are known to lack DNMT1, 3A and 3B and appear to possess DNMT2 as the only known DNA methyltransferase (Glastad, Hunt & Goodisman, 2014). Despite reports on the presence of DNA methylation in solitary or non-social insects like stick insects and moths; not much is known about its role and importance. The exact involvement of genomic methylation in molecular processes of *Drosophila*, if any, remains unexplored.

## Conclusion

Our analysis of the complete life cycle of *D. melanogaster* and eleven other species conclusively establishes the presence of DNA methylation in the members of genus *Drosophila*. This modification undergoes change during holometabolous development. Also, it suggests that there is no effect from factors like sex, phylogenetic relatedness and genome GC content on the levels of 5mC in *Drosophila*. Despite the absence of typical (DNMT1 and DNMT 3A/3B) DNA methyltransferases, there is DNA methylation in all twelve species of genus *Drosophila*. These findings pose an interesting problem that demand further investigation which can lead to the identification of a methyltransferase enzyme with novel functions.

##  Supplemental Information

10.7717/peerj.5119/supp-1 Supplemental Information 1 Nucleotide counting code

10.7717/peerj.5119/supp-2 Figure S1 Difference in the levels of 5mC in head tissue and the whole body of adult male and female of *Drosophila melanogaster* * and ++ indicate significance of comparisons (*α* = 0.05, *p* = 0.0013, Kruskal–Wallis test). All error bars represent SEM.

10.7717/peerj.5119/supp-3 Figure S2 The neighbour joining tree The original NJ tree with the scale for the twelve members of genus *Drosophila.*

10.7717/peerj.5119/supp-4 Figure S3 Divergence time and DNA methylation of genus *Drosophila* The divergence time and levels of 5mC of the twelve members of genus *Drosophila.*

10.7717/peerj.5119/supp-5 Data S1 Supplementary data

## Additional Information and Declarations

### Data Availability

The following information was supplied regarding data availability: The raw data are provided in the Supplemental Files.
