# Supplementary figures and images for: Levels of DNA cytosine methylation in the Drosophila genome
Source: PeerJ. 2018 Jul 2;6:e5119. doi: 10.7717/peerj.5119 (PMC6033079; doi:10.7717/peerj.5119)

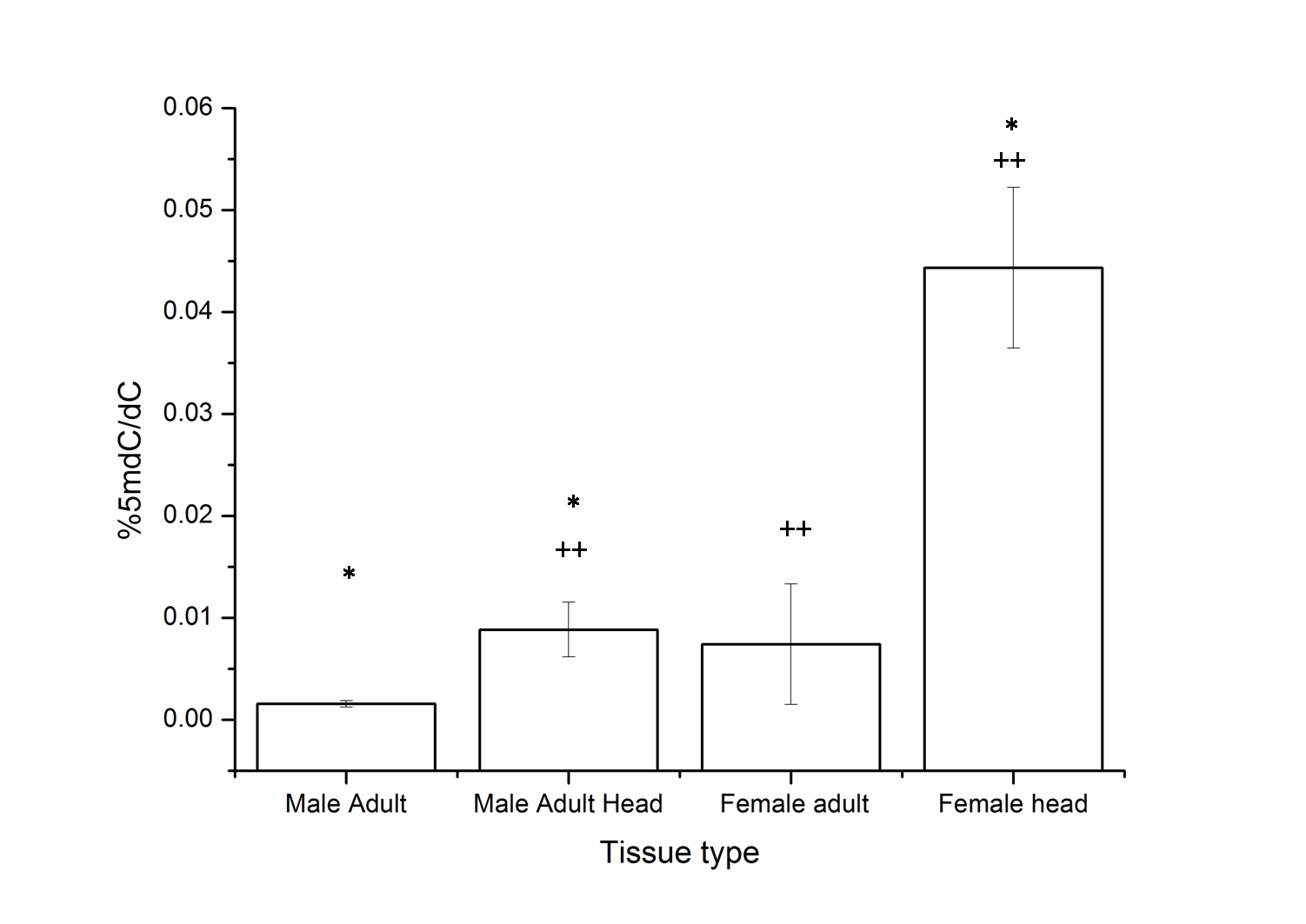

Supplement: Figure S1 — * and ++ indicate significance of comparisons (α = 0.05, p = 0.0013, Kruskal–Wallis test). All error bars represent SEM. [file peerj-06-5119-s002.png]

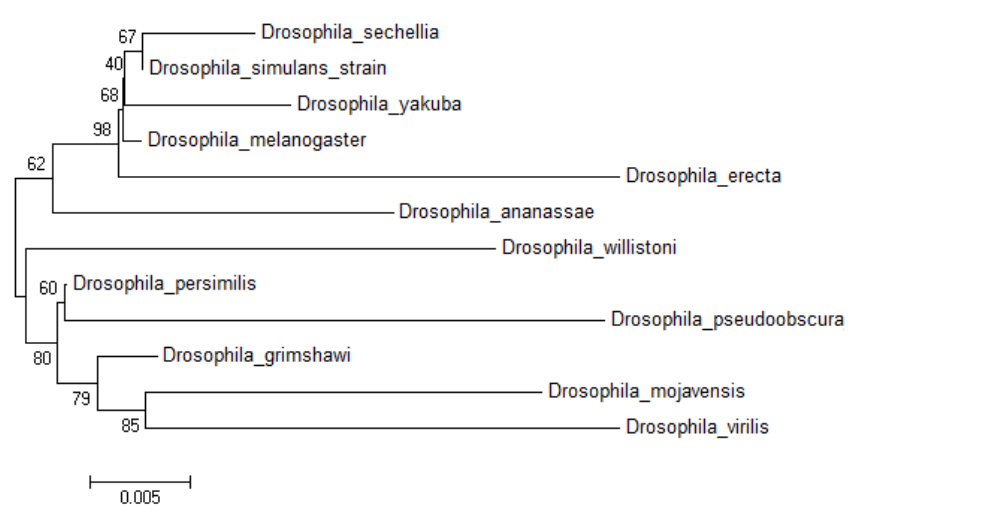

Supplement: Figure S2 — The original NJ tree with the scale for the twelve members of genus Drosophila. [file peerj-06-5119-s003.png]

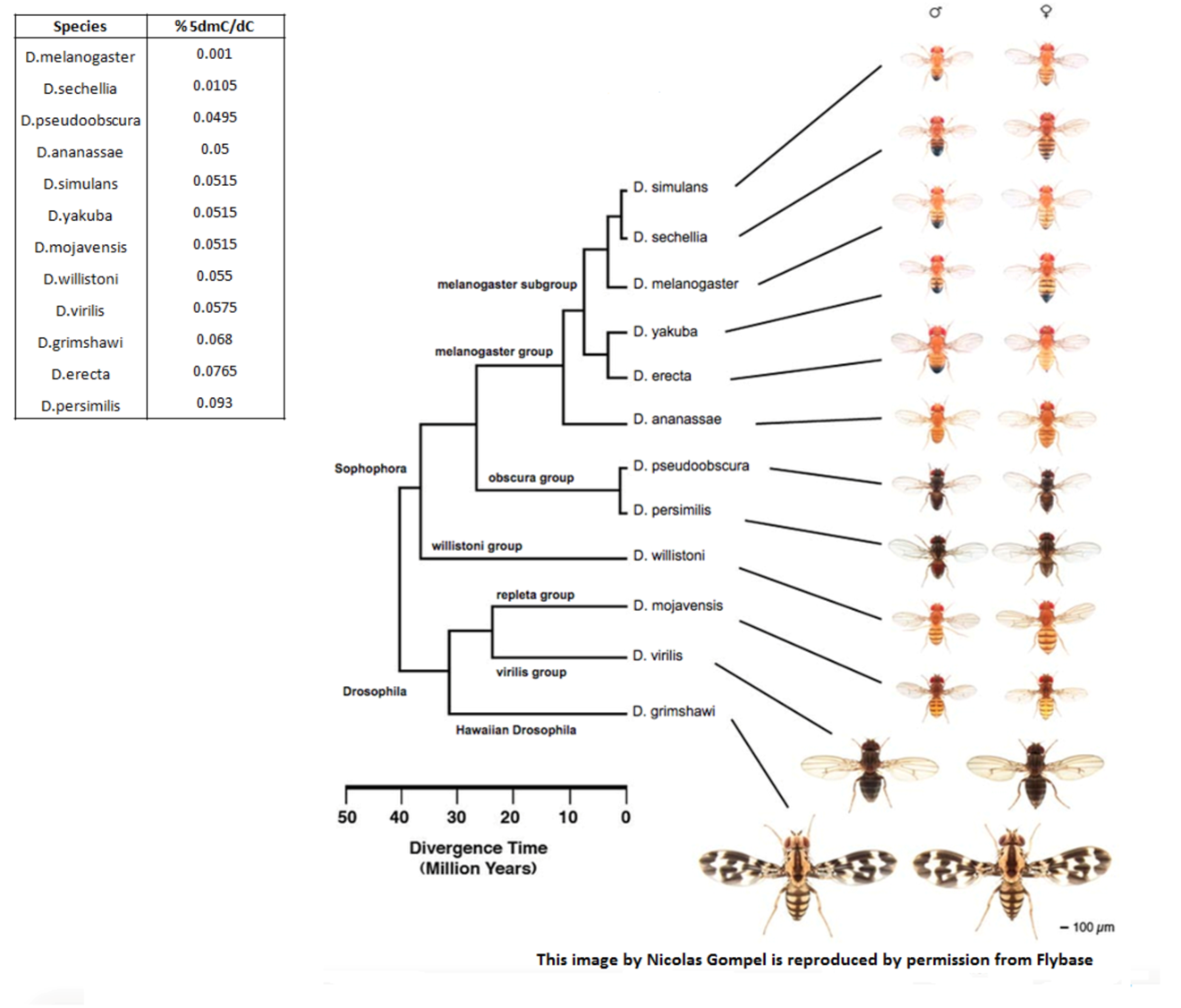

Supplement: Figure S3 — The divergence time and levels of 5mC of the twelve members of genus Drosophila. [file peerj-06-5119-s004.png]
